# Psychiatric treatment of mentally ill persons in custody – legal, medical and ethical issues

**DOI:** 10.1192/j.eurpsy.2021.1018

**Published:** 2021-08-13

**Authors:** V. Sendula Jengic, A. Jengic Bujan

**Affiliations:** 1 Department For Forensic Psychiatry, Rab Psychiatric Hospital, Rab, Croatia; 2 Jengic Bujan, Law Office, Rijeka, Croatia

**Keywords:** forensic psychiatry, pre-trial detention, Mental Health Act, hospital mental health services

## Abstract

**Introduction:**

The most recent legal regulations in the Republic of Croatia govern the process of criminal procedure for persons in pre-trial detention who have a temporary mental disturbance for which psychiatric treatment is needed. The Prison Director is in this case obliged to seek psychiatric treatment for such persons who are then hospitalized in a psychiatric institution instead of a prison hospital or prison that meets the requirements prescribed by law for the accommodation of pre-trial detainees. Forensic departments of the five psychiatric hospitals in Croatia accept mentally incapable persons subject to court order, but not persons in custody, i.e. in pre-trial detention. Pre-trial detention is a measure imposed in the previous proceedings to ensure that the person to whom the measure is imposed is present during the pre-trial stage and the hearing stage, i.e. after the final judgment has been rendered until it becomes final. According to Croatian laws, a person who has been sentenced to pre-trial detention and who has mental disorders is entitled to a range of rights that must be respected, and at the same time, there are strict restrictions in exercising those same rights for the reason of sentencing to pre-trial detention.

**Objectives:**

The article points to several problems that have arisen in practice due to the under-regulation of pre-trial detention measures.

**Methods:**

Perspective, opinion, and commentary article.

**Results:**

Perspective, opinion, and commentary article.

**Conclusions:**

The authors discuss legal, medical, and ethical issues, but also the financial framework of such a process.

